# Insulin-Like Growth Factor Binding Protein-4 as a Marker of Chronic Lupus Nephritis

**DOI:** 10.1371/journal.pone.0151491

**Published:** 2016-03-28

**Authors:** Tianfu Wu, Chun Xie, Jie Han, Yujin Ye, Sandeep Singh, Jinchun Zhou, Yajuan Li, Huihua Ding, Quan-zhen Li, Xin Zhou, Chaim Putterman, Ramesh Saxena, Chandra Mohan

**Affiliations:** 1 Department of Biomedical Engineering, University of Houston, Houston, Texas, United States of America; 2 Division of Rheumatology, The University of Texas Southwestern Medical Center, Dallas, Texas, United States of America; 3 Division of Nephrology, The University of Texas Southwestern Medical Center, Dallas, Texas, United States of America; 4 Department of Immunology, The University of Texas Southwestern Medical Center, Dallas, Texas, United States of America; 5 Baylor University Medical Center at Dallas, Dallas, Texas, United States of America; 6 Division of Rheumatology, Albert Einstein College of Medicine, Bronx, New York, United States of America; UNIFESP Federal University of São Paulo, BRAZIL

## Abstract

Kidney biopsy remains the mainstay of Lupus Nephritis (LN) diagnosis and prognostication. The objective of this study is to identify non-invasive biomarkers that closely parallel renal pathology in LN. Previous reports have demonstrated that serum Insulin-like growth factor binding protein 4 (IGFBP-4) was increased in diabetic nephropathy in both animal models and patients. We proceeded to assess if IGFBP4 could be associated with LN. We performed ELISA using the serum of 86 patients with LN. Normal healthy adults (N = 23) and patients with other glomerular diseases (N = 20) served as controls. Compared to the healthy controls or other glomerular disease controls, serum IGFBP-4 levels were significantly higher in the patients with LN. Serum IGFBP-4 did not correlate well with systemic lupus erythematosus disease activity index (SLEDAI), renal SLEDAI or proteinuria, but it did correlate with estimated glomerular filtration rate (*R* = 0.609, *P* < 0.0001). Interestingly, in 18 patients with proliferative LN whose blood samples were obtained at the time of renal biopsy, serum IGFBP-4 levels correlated strongly with the chronicity index of renal pathology (*R* = 0.713, *P* < 0.001). IGFBP-4 emerges a potential marker of lupus nephritis, reflective of renal pathology chronicity changes.

## Introduction

Systemic lupus erythematosus (SLE) is a systemic autoimmune disorder affecting multiple organ systems. Renal involvement remains the leading cause of mortality and morbidity in SLE, despite intensive systemic immunosuppressive therapies.[1–6] Early diagnosis and prompt treatment of lupus nephritis (LN) are associated with significantly better outcome.[7–11]

Current laboratory tests for lupus nephritis such as proteinuria, serum creatinine, titers of autoantibodies, and complement levels lack sensitivity and specificity for characterizing or predicting the underlying renal damage.[12] The kidney biopsy remains the gold standard for providing information on the relative degree of activity and chronicity of disease in the kidneys, and is of pivotal importance in prognostication and in guiding therapy. In particular, the chronicity index on renal pathology is highly predictive of renal and patient mortality.[7, 11, 13, 14]. Even in patients without clinical manifestations of renal disease, and in those with only mild proteinuria, the frequencies of proliferative LN are surprisingly high.[15, 16] Furthermore, nephritic flares are not uncommon in SLE, and are associated with poor prognosis.[17–20] Nephritic flares may sometimes suggest transformation from one histologic pattern to another.[21] In addition, distinction between a nephritic flare and chronic renal damage could be difficult.[22] Therefore, a repeat biopsy may be necessary in certain circumstances to guide the decision on immunosuppressive therapy.[21–23] Since renal biopsy is invasive and associated with significant risk, there is an urgent need for the identification of non-invasive, surrogate biomarkers that closely parallel renal pathology.

Indeed, great effort has been expended in the past few years to identify biomarkers reflecting various aspect of renal disease in SLE. Several recent studies [24–27] and reviews [28–30] have revealed a dozen of urine and serum biomarkers that could potentially predict LN activity, renal flare and most importantly, renal pathology. Thus far, most of the studies have focused on identifying biomarkers that reflect renal disease activity. Little has been reported on biomarkers that are indicative of chronicity changes in LN.

In this study we found Insulin-like growth factor binding protein-4 (IGFBP-4) was significantly elevated in lupus nephritis, particularly those with renal pathology chronicity changes.

## Patients and Methods

### Patients

In this cross-sectional study, patients with biopsy proven LN and controls were recruited from the renal clinic at Parkland Hospital and St. Paul University Hospital, both affiliated with the University of Texas Southwestern Medical Center, Dallas, Texas, between 2007–2011. All human related studies were conducted in accordance with institutional review board approved protocols at UT Southwestern Medical Center, Dallas, TX. Blood and urine samples as well as clinical data were collected at the time of visit. Sera and urine samples were stored in aliquots at −80°C until use. Serum samples from an independent cohort of 86 biopsy-proven LN patients were used for validation studies. Lupus nephritis was diagnosed and classified based upon ISN/RPS 2003 classification. Inclusion criteria included LN patients with biopsy-proven LN. Exclusion criteria were patients with end-stage renal disease. Clinical data was gathered by chart review, and SLEDAI was calculated based on chart review. The characteristics of 86 LN patients used for the initial study are listed in Table 1. Gender and age-matched healthy volunteers (n = 23) were recruited as healthy controls. In addition, 20 patients with other glomerular diseases (referred to as chronic kidney disease, CKD) were recruited as disease controls. The characteristics of the healthy subjects and disease controls are listed in Table 2. Finally, serum from 15 patients with Rheumatoid Arthritis (RA) were obtained from Dr. Putterman and tested as controls.

**Table 1:** Demographics and Clinical characteristics of LN patients used for validations studies.

No.	86
Female, no.	74
Age, years, mean +/- SE	35.0 +/- 1.2
Race, African American/Hispanic/Caucasian/Asian, no.	34/43/6/3
SLEDAI, median (interquartile)	9.5 (4.0–14.5)
Renal SLEDAI, median (interquartile)	4.0 (4.0–8.0)
No. of patients with active renal disease, no. (%)	68 (79.1)
Renal Pathology	
ISN/RPS classification	
II	12
III	21
IV	35
V	18
Urine protein:Cr ratio, mg/mg, mean +/- SE	1.27 +/- 0.16
Serum Cr, mg/dl, mean +/- SE	1.46 +/-0.13
Current medications, no.	
Prednisone	61
Mycophenolic acid	26
Azathioprine/MTX	11
Cyclophosphamide	8
Cyclosporine/Tacrolimus	2
Hydrochloroquine	34
Angiotensin blocking agents	40

Cr, creatinine; MTX, methotrexate; SE, standard error; SLEDAI, systemic lupus erythematosus disease activity index.

**Table 2:** Characteristics of Controls used for validation studies.

	CKD	Healthy
No.	20	23
Age, years, Mean +/- SE	54.2+/- 1.9	34.6 +/- 1.8
Female/male	15/5	14/9
Race, Black/Hispanic/Caucasian/Asian	4/8/6/2	9/9/3/2
Scr, mg/dl, mean +/- SE	1.78 +/- 0.23	0.84 +/- 0.05
Urine protein:Cr ratio, mg/mg, mean +/- SD	1.97 +/- 0.42	0.10 +/- 0.01
Primary disease		
Minimal change disease	2	N/A
Membranous nephropathy	3	N/A
FSGS	2	N/A
ANCA-associated GN	2	N/A
Diabetic nephropathy	12	N/A

ANCA, anti-neutrophil cytoplasmic antibody; CKD, chronic kidney disease; Cr, creatinine; FSGS, focal segmental glomerulosclerosis; GN, glomerulonephritis; SE, standard error.

Systemic lupus erythematosus disease activity index (SLEDAI) scores at the time of sample collection were calculated based on chart review, as described previously.[31] Also documented was the renal related SLEDAI (rSLEDAI) that consists of the four kidney-related criteria (i.e., hematuria, pyuria, proteinuria, and urinary casts) of the SLEDAI.[31]

### Renal pathology

Renal pathology was assessed by a renal pathologist, using International Society of Nephrology/Renal pathological society (ISN/RPS) classification.[32] For those with proliferative lupus nephritis (ISN/RPS class III or class IV), the activity index (AI) and chronicity index (CI) were also calculated using a well-established scoring system [13, 24]. In brief, for the Activity Index (AI), each specimen was assessed for 6 components: cellular crescents, fibrinoid necrosis or karyorrhexis, endocapillary proliferation, leukocytic infiltration, hyaline thrombi or wire loops, and interstitial infiltration. Each feature was scored on a scale of 0 to 3, except for fibrinoid necrosis and crescents, which were weighted twice. Chronicity Index (CI) was scored based on the presence of glomerular sclerosis, fibrous crescents, interstitial fibrosis and tubular atrophy, also on a scale of 0 to 3. Therefore, the maximum possible AI score is 24, and the maximum possible CI score is 12.

### Serum collection

Whole blood was collected in BD Vacutainer Serum tubes (Cat #: 367812). Tubes were incubated undisturbed at room temperature for 20 min, and then centrifuged at 3,000 rpm for 10 min at 4°C. The supernatant (serum) was divided into 200-uL aliquots and frozen at -80°C for storage. Each aliquot of serum was retrieved and thawed only once for the assays in this study.

### ELISA

In the validation study, serum IGFBP-4 concentrations were measured using a human IGFBP-4 ELISA kit from R&D Systems (Minneapolis, MN), according to the manufacturer’s manual. Briefly, 96-well microplates were coated with the capture antibody overnight. After washing and blocking, 1:100 diluted sera were added. After reaction, the detection antibody was added, followed by streptavidin-HRP, and substrate, sequentially. The optical density was then read with a microplate reader ELX808 from BioTek Instruments (Winooski, VT). Serum IGFBP-4 concentrations were calculated from a standard curve. Urine IGFBP-4 was also measured using the same kit. Urinary IGFBP-4 was normalized against urine creatinine concentration.

### Serum creatinine, urine protein and creatinine detection

Urinary protein concentrations were determined using the Coomassie Plus protein assay kit (Pierce, Rockford, IL). Urinary and serum creatinine concentrations were determined with a VITROS 250 automatic analyzer. Estimated glomerular filtration rate (eGFR) was calculated using the MDRD Study equation.[33]

### Statistics

Data were analyzed and plotted using the GraphPad Prism 5 software (GraphPad, San Diego, CA). For comparison between two groups, a t-test with Welch’s correction was used where the normality test passed; otherwise, the nonparametric Mann-Whitney test was used to analyze the data. For correlation analysis, the Pearson method or the nonparametric Spearman method was used.

## Results

### Increased serum IGFBP-4 levels in patients with lupus nephritis

**Fig 1:**
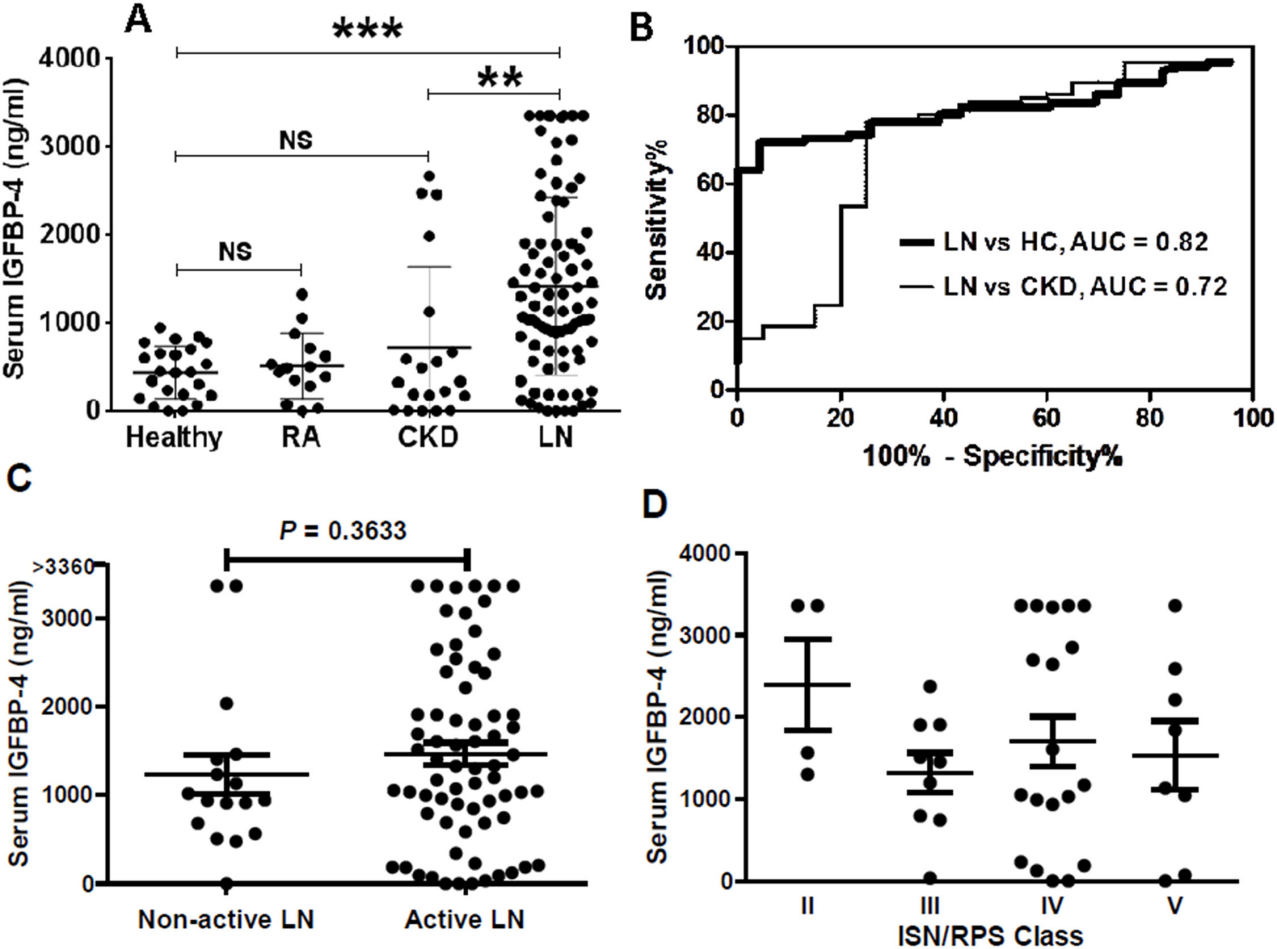
Serum IGFBP-4 levels were elevated in patients with lupus nephritis. Serum 86 patients with LN, 23 healthy controls, 20 patients with CKD and 15 patients with RA were assayed for serum IGFBP-4 levels by ELISA. Plotted in **(A)** are serum levels of IGFBP-4 in the 3 groups, indicating significantly higher levels in LN as compared to the CKD, RA and healthy controls (*P* < 0.01 and *P* < 0.0001, respectively). Receiver operating characteristic curves were generated using serum IGFBP-4 levels measured by ELISA **(B)**. Also indicated are the corresponding AUC values. Plotted in **(C)** are serum IGFBP-4 levels in two groups of patients with LN, those with active LN (rSLEDAI > 0) and those with non-active LN (rSLEDAI = 0), *P* > 0.3. **D**: serum IGFBP-4 levels in patients with LN of different ISN/RPS classes, whose blood samples were obtained within 5 months of renal biopsy. No statistical significance were found among the patients with LN of classes II (n = 4), III (n = 9), IV (n = 19), and V (n = 8), *P* > 0.05.

**Fig 2:**
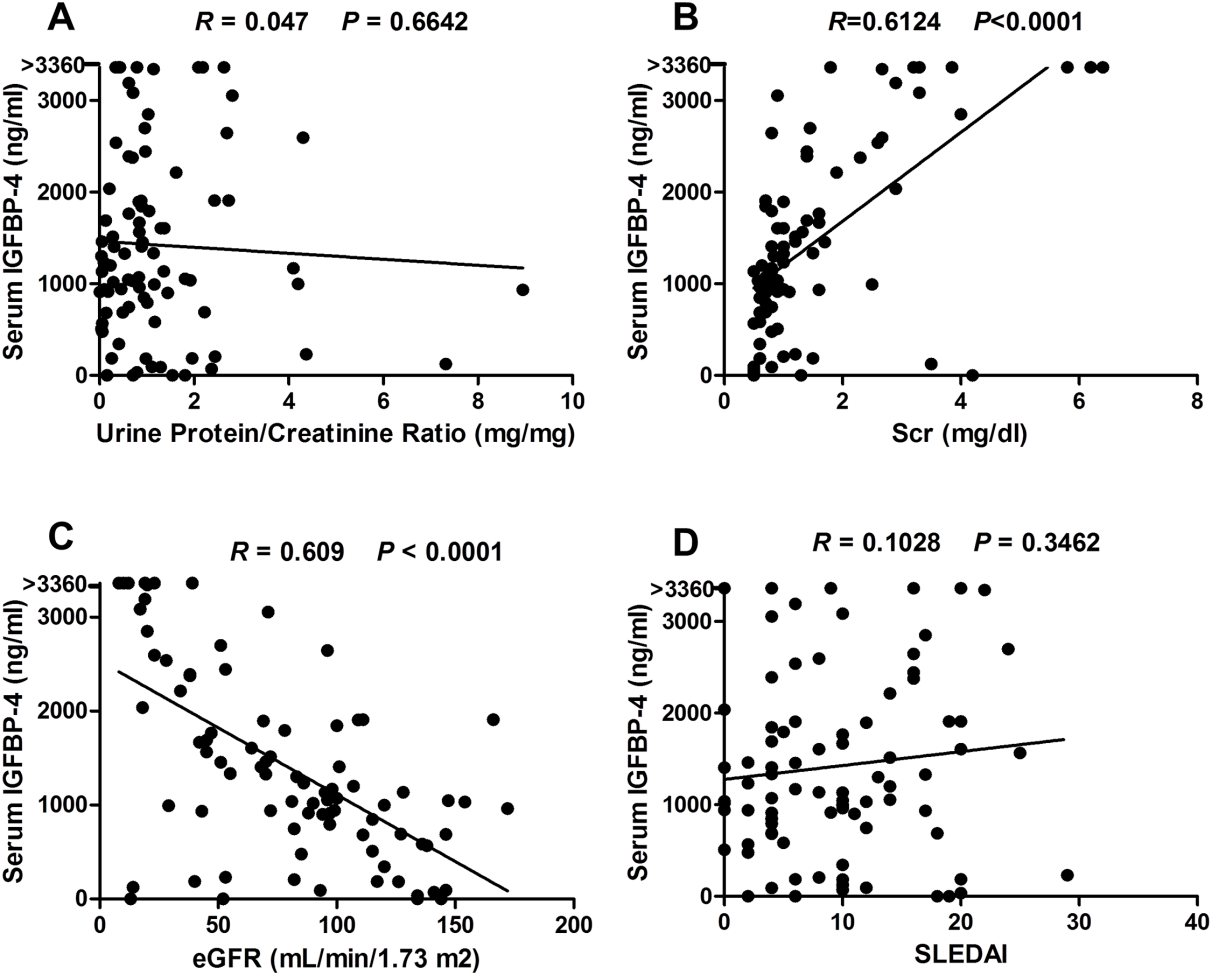
Correlation between serum IGFBP-4 levels and clinical parameters. Serum IGFBP-4 levels were plotted against severity of proteinuria, as measured by spot urinary protein:creatinine ratio (**A**), serum creatinine (**B**), eGFR (as calculated using the MDRD study equation (**C**), and systemic lupus erythematosus disease activity index (SLEDAI, **D**), respectively. Also plotted are the corresponding *R* and *P* values. eGFR, estimated glomerular filtration rate.

Serum IGFBP-4 concentrations were measured using ELISA, in a cohort of LN patients and in healthy controls. Serum IGFBP-4 levels was significantly elevated in patients with LN (mean ± SE, 1422 ± 109 ng/ml), as compared to healthy controls (442 ± 62 ng/ml, *P* < 0.001, Fig 1A). To determine if IGFBP-4 is a relatively specific marker for LN, serum IGFBP-4 concentrations from a group of patients with other chronic kidney diseases (CKD) and rheumatoid arthritis (RA) were also determined. The primary diseases among the CKD controls included minimal change disease, membranous nephropathy, focal segmental glomerulosclerosis (FSGS), diabetic nephropathy and anti-neutrophil cytoplasmic antibody (ANCA)-associated glomerulonephritis (Table 2). Indeed, serum IGFBP-4 levels were significantly higher in patients with LN, as compared to that of patients with other CKD (725 ± 204 ng/ml, *P* < 0.01, Fig 2A). Interestingly, although some of the CKD patients had increased serum IGFBP-4 levels, the mean serum IGFBP-4 levels in CKD patients were not significantly higher than that in healthy controls (*P* > 0.1). Likewise, the serum concentrations of IGFBP-4 in RA patients were similar to that in healthy controls (Fig 1A).

To examine how well serum IGFBP-4 performs in discriminating LN patients from healthy controls and from the CKD controls, a receiver operating characteristic (ROC) was constructed. As shown in Fig 1B, the serum levels of IGFBP-4 exhibited relatively good ROC profiles, with area under curve (AUC) values of 0.82 (*P* < 0.0001) and 0.72 (*P* = 0.0019), respectively, reflecting degrees of sensitivity and specificity for distinguishing SLE patients from healthy controls and from CKD controls.

We then examined if serum IGFBP-4 can distinguish patients with active LN from those with non-active LN. Non-active LN is defined as rSLEDAI of 0, while active LN is defined as rSLEDAI > 0. In our cohort of 86 biopsy-proven LN patients, 17 were found to be non-active in renal disease at the time of sampling. As shown in Fig 1C, serum IGFBP-4 levels did not differentiate those with active renal disease from those with non-active renal disease (1469 ± 124 vs 1232 ± 223 ng/ml, *P* = 0.363).

As the LN patients exhibited a variety of classes of renal pathology, we further analyzed if serum IGFBP-4 levels differed among patients with various ISN/RPS pathological classes. For this sub-analysis, we focused our analysis on the data from 40 patients whose blood samples were obtained within 5 months of their renal biopsy. As illustrated in Fig 1D, there were no statistically significant differences in serum IGFBP-4 levels among patients with LN class II, III, IV, V (*P* > 0.05).

### Correlation of IGFBP-4 with clinical parameters

To explore the possibility of using serum IGFBP-4 levels to predict clinical features of LN, particularly disease activity, correlation analyses were performed. Although serum IGFBP-4 levels correlated poorly with severity of proteinuria, as indicated by urinary protein:creatinine ratio (*R* = 0.047, *P* > 0.6, Fig 2A), they correlated positively with serum creatinine levels (*R* = 0.612, *P* < 0.0001, Fig 2B), and inversely with eGFR, as calculated using the MDRD Study equation (*R* = -0.609, *P* < 0.0001, Fig 2C), in patients with LN. However, serum IGFBP-4 levels did not correlate with SLE disease activity (SLEDAI, *R* = 0.1028, *P* = 0.3462, Fig 2D), nor with rSLEDAI (*R* = 0.0613, *P* = 0.575). There was a trend suggesting that IGFBP-4 may correlate with Scr (*R* = 0.396, *P* = 0.09) among the CKD controls.

### Correlation of IGFBP-4 levels with concurrent renal pathology

**Fig 3:**
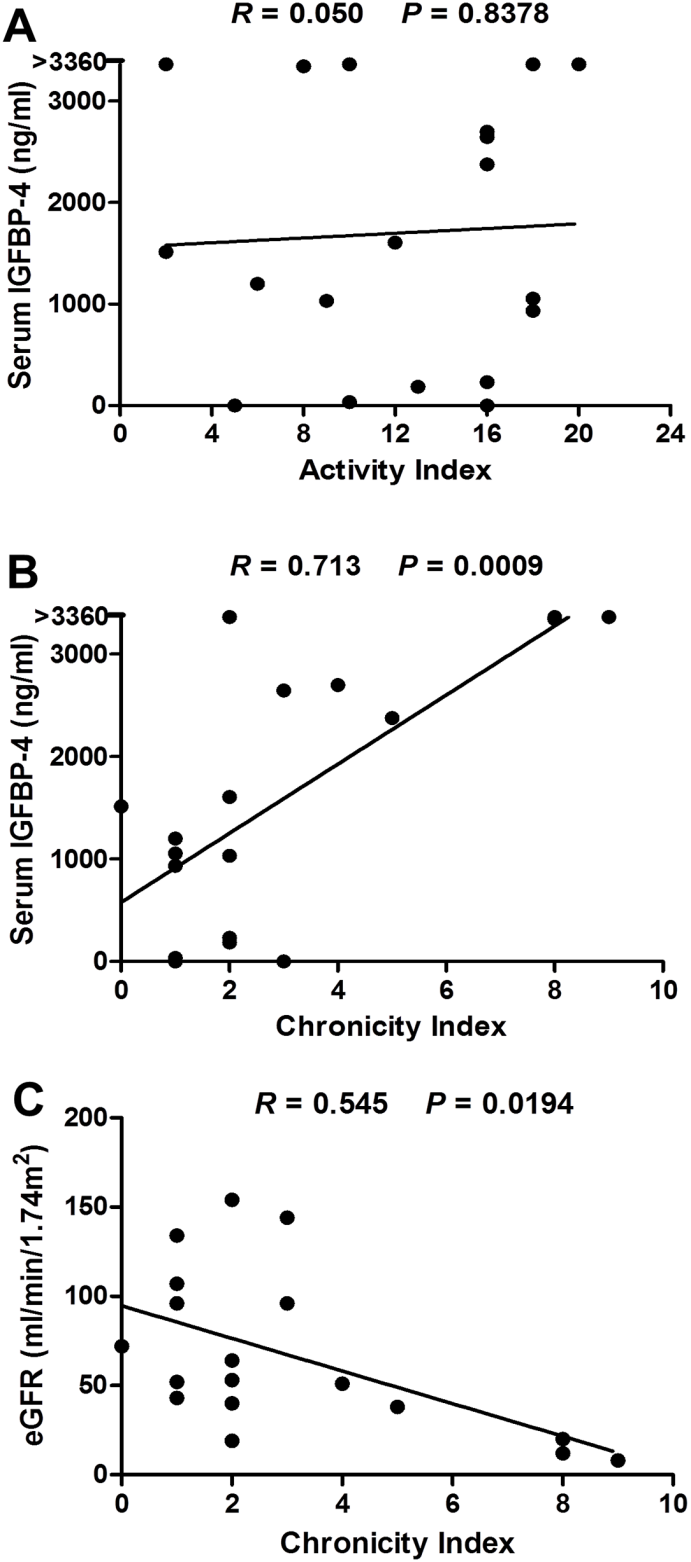
Correlation between serum IGFBP-4 levels with renal pathology. Serum IGFBP-4 levels in 18 patients with proliferative (ISN/RPS class III and class IV) LN whose blood samples were obtained at the time of renal biopsy were correlated with the activity index (**A**) and chronicity index (**B**) of renal pathology, as detailed in methods. Also depicted are correlations of eGFR with chronicity index (**C**). eGFR, estimated glomerular filtration rate.

As serum IGFBP-4 correlated with eGFR in patients with LN, and since it has been reported that IGFBP-4 is increased in patients with chronic renal failure, [34, 35] we next explored if elevated serum IGFBP-4 is indicative of chronic lupus nephritis. For this purpose, we focused on a unique subset of patients whose serum samples were obtained at the time of kidney biopsy. Thus, in 18 patients with proliferative LN (3 with ISN/RPS class III and 15 with class IV), their blood samples were obtained on the day of renal biopsy. The renal pathology activity index and chronicity index in these patients range from 2–20 and 0–9, respectively. Interestingly, serum IGFBP-4 levels correlated strongly with the chronicity index of renal pathology (*R* = 0.713, *P* < 0.001, Fig 3B) but not with the activity index (*R* = 0.05, *P* > 0.80, Fig 3A). Indeed, serum IGFBP4 was better correlated with the chronicity index than eGFR (Fig 3C). Again, serum IGFBP-4 levels did not discriminate patients with class III LN from those with class IV LN (data not shown), in this subset of patients. In contrast to the serum, minimal or undetectable IGFBP-4 levels were found in the urine samples from patients with LN, as well as from CKD and healthy controls (data not shown).

Serum IGFBP-4 levels did not correlate with the treatment the patient was on. Thus, the patients who were on steroids (N = 61), Cytoxan (N = 8), MMF (N = 26), azathioprine (N = 9), methotrexate (N = 11), Plaquenil (N = 34), ACE inhibitors or ARB (N = 40) exhibited mean serum IGFBP-4 levels of 1458, 854, 1183, 1639, 1448, 1121 and 1437 ng/ml, respectively, and none of these values were significantly different from the serum IGFBP-4 levels in SLE patients not on any medication (N = 9; mean = 1808 ng/ml). Although serum IGFBP-4 levels did not correlate with complement levels, a weak correlation was noted with anti-dsDNA antibodies (correlation coefficient = 0.23, p = 0.048).

**Fig 4:**
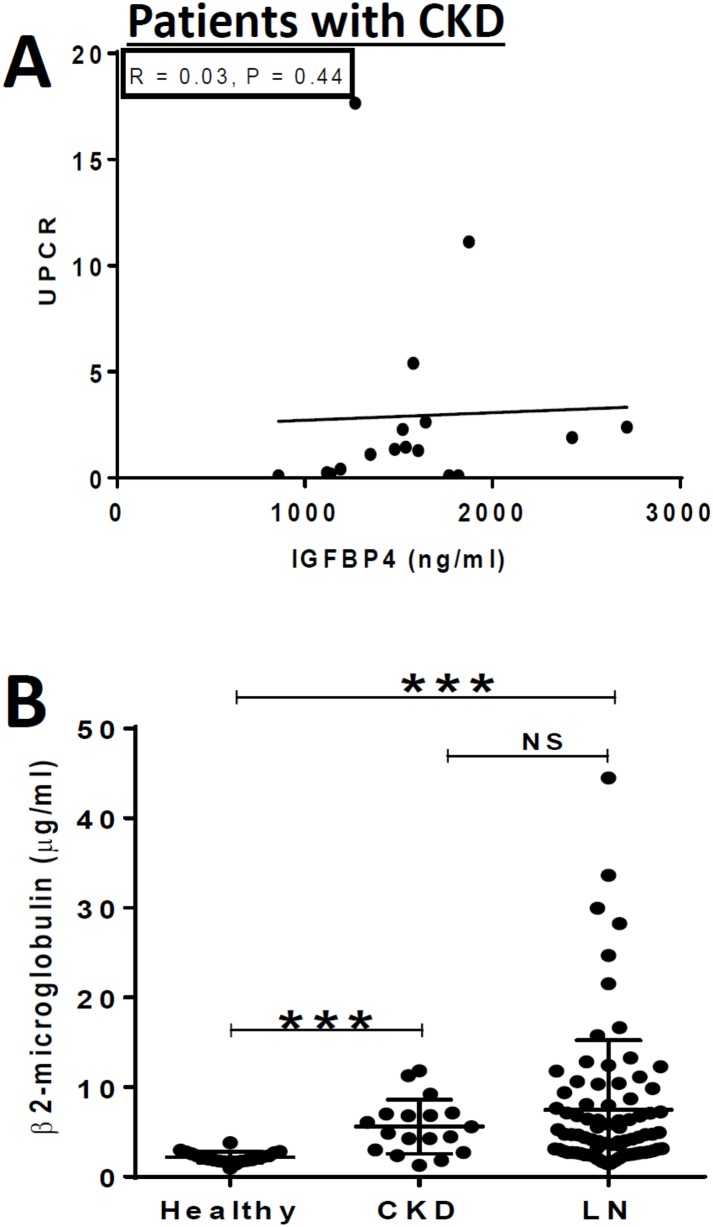
Plotted in **(A)** are urine protein creatinine ratios (UPCR) versus serum IGFBP4 levels in the CKD control subjects. Plotted in **(B)** are the serum beta-2-microglobulin levels, as assessed by ELISA, in lupus nephritis patients, healthy controls and CKD controls. Other details are as listed in Fig 1 legend.

We next asked if the differences seen in serum IGFBP-4 levels in different subjects with renal disease might be affected by differences in the degree of proteinuria. However, in both the CKD patients and in patients with LN, serum IGFBP-4 levels were not associated with the degree of proteinuria, as depicted in Figs 2A and 4A. We also identified a group of LN patients and CKD controls (N = 17 each) with similar mean urine protein creatinine ratios (UPCR = 1.89). Even in these two proteinuria-matched groups, the IGFBP4 levels were significantly different; in the CKD control group, the mean IGFBP4 level was 737.4 ng/ml, whereas in the LN group the mean IGFBP4 level was 1477.6 ng/ml (p< 0.014), indicating that the increased IGFBP4 in LN was not a consequence or reflection of proteinuria.

IGFBP4 is a relatively small protein with a molecular weight of 34 kD. We next tested the alternate hypothesis that the observed increase in serum IGFBP4 may not be specific or restricted to IGFBP4, but may be reflective of a generalized increase in other molecules of similar molecular weight, possibly because of reduced glomerular filtration rates in these patients. For this purpose, we tested the levels of serum beta-2-microglobulin, another small protein of 11kD molecular weight. Although serum levels of this molecule were similar in both the LN patients and CKD controls, both of these groups had significantly higher levels of beta-2-microglobulin, compared to the healthy controls (Fig 4B). Clearly, a more comprehensive study of other serum proteins has to be executed to fully address this important question.

## Discussion

Based on an initial array-based screen, serum IGFBP-4 emerged as a potential marker of LN. This was confirmed with another platform in a larger cohort of LN patients. Furthermore, serum IGFBP-4 levels appear to be able to discriminate LN patients from healthy volunteers and from patients with other glomerular diseases. Most importantly, we demonstrate that serum IGFBP-4 correlates strongly with the chronicity of renal pathology in LN patients. The CKD control patients were similar to LN patients in the degree of proteinuria (*P* > 0.05) and in their levels of serum creatinine (*P* > 0.05). Although it has been reported that circulating IGFBP-4 levels increase slightly with age in healthy female adults, [36] we did not detect any age-, gender- or ethnic-differences in serum IGFBP-4 concentrations among the lupus patients and controls (data not shown). We did not note any difference in serum IGFBP-4 levels between patients on immunosuppressants compared to those who were not. Likewise, the taking angiotensin blocking agents or hydrochloroquine did not appear to affect serum IGFBP-4 levels among LN patients (data not shown). Finally, serum IGFBP-4 levels were not associated with any particular co-morbidity.

Perhaps the most important finding from the current study is that circulating IGFBP-4 levels correlate well with chronicity of renal pathology. It has been shown in several studies that high chronicity index in renal pathology is associated with poor clinical outcome.[7, 11, 13, 14, 37] A few studies have correlated some of the markers with histologic findings of LN [24, 38–43] mostly with LN classes. In clinical practice, it is critical to distinguish ongoing inflammation from chronic fibrosis, as the former warrants aggressive immunosuppressive therapy, whereas the risk of using potential toxic immunosuppressant may outweigh the benefit in patients with extensive chronic renal disease. Thus, while it is extremely important to identify blood or urine biomarkers that are indicative of inflammation and ongoing autoimmune activity, biomarkers of irreversible damage would also be very helpful in guiding clinical decision. It has long been recognized that serum creatinine is not a reliable indicator of renal function in patients with LN. GFR, usually estimated from serum creatinine based equations, when decreased, may reflect chronic renal failure. Acute inflammation in the kidneys in LN patients, on the other hand, may also cause a decrease in GFR. This is evidenced by the fact that eGFR not only correlated with the CI but also with the AI to some extent, among the LN patients. Thus far, little has been reported regarding potential markers predicting chronicity of LN.[38, 44] The few reports that have attempted to do so involved relatively complex technologies or required renal tissues, and hence may not be practical for daily clinical practice. In this regard, serum IGFBP-4 performs better than eGFR in predicting chronicity of renal disease (Fig 3).

As with previous biomarkers described in the field, serum IGFBP-4 is not perfect in predicting renal pathology (*R* = 0.713). Nevertheless, taken together with previously identified activity markers, serum IGFBP-4 may substantially improve the clinicians’ ability to predict renal prognosis and hence may be helpful in guiding individualized therapy for LN, without incurring the risks of renal biopsy. For instance, in a LN patient with high serum IGFBP-4 but low levels of activity, immunosuppressive therapy may do more harm than benefit to the patient, and therefore, conservative treatment may be a better choice. Alternatively, approaches to reverse chronicity changes may be instituted.

IGFBP-4 is a member of the insulin-like growth factor binding protein family. The protein binds both insulin-like growth factors (IGFs) I and II, and regulates their function by altering their interaction with IGF receptors and prolonging the half-life of the IGFs. It plays an important role in reproductive physiology, bone formation, and growth regulation of cancer.[45, 46] IGFBP-4 is widely expressed in vivo and has been identified in all biological fluids. In particular, IGFBP-4 is abundantly expressed in the kidney in humans and in animals.[47–49] It has been reported that elevation of IGFBP-4 levels in serum correlates moderately with the degree of chronic renal dysfunction in adults and in children (35,36). In resonance with these findings, in our study, serum IGFBP-4 levels also correlated with eGFR in LN patients. Since the antibodies used in the IGFBP4 ELISA were raised against a recombinant IGFBP4-derived peptide, it also remains to be stablished if the molecule detected in our study is intact IGFBP4 or fragments derived from it.

One limitation of the current study is the limited numbers of patients, particularly the ones from whom we have concurrent serum/pathology specimens. Ongoing recruitment efforts are aimed at boosting these patient numbers. This study opens up several new questions that need to be addressed. Currently, we do not know if elevated serum IGFBP-4 in patients with LN is due to increased production, decreased degradation, or impaired glomerular filtration of this molecule. Another parallel study in our laboratory that entailed a comprehensive scan of the serum levels of 1000 different proteins in LN did not reveal a relationship between the molecular weight of the protein and the serum level of the protein in LN (manuscript in preparation). Hence, it seems unlikely that the elevation in serum IGFBP4 in LN is solely the consequence of impaired eGFR. Nevertheless, it would be important to identify additional serum proteins that may offer complementary information, in an effort to assemble biomarker panels that are more predictive of clinical and pathological disease progression in LN. It is also important to execute longitudinal studies to establish if serum IGFBP-4 can indeed predict future disease progression in LN. As IGFBP-4 is an important regulator of the IGF system, it will be very interesting to determine whether IGFBP-4 and IGFs play a role in the pathogenesis of renal fibrosis. Whether this axis can be therapeutically targeted is also an open question.
